# Crystal structure of mimivirus uracil-DNA glycosylase

**DOI:** 10.1371/journal.pone.0182382

**Published:** 2017-08-01

**Authors:** Eunju Kwon, Deepak Pathak, Hyeun Wook Chang, Dong Young Kim

**Affiliations:** College of Pharmacy, Yeungnam University, Gyeongsan, Gyeongbuk, South Korea; Saint Louis University, UNITED STATES

## Abstract

Cytosine deamination induced by stresses or enzymatic catalysis converts deoxycytidine into deoxyuridine, thereby introducing a G to A mutation after DNA replication. Base-excision repair to correct uracil to cytosine is initiated by uracil-DNA glycosylase (UDG), which recognizes and eliminates uracil from DNA. Mimivirus, one of the largest known viruses, also encodes a distinctive UDG gene containing a long N-terminal domain (N-domain; residues 1–130) and a motif-I (residues 327–343), in addition to the canonical catalytic domain of family I UDGs (also called UNGs). To understand the structural and functional features of the additional segments, we have determined the crystal structure of UNG from *Acanthamoeba polyphaga* mimivirus (mvUNG). In the crystal structure of mvUNG, residues 95–130 in the N-domain bind to a hydrophobic groove in the catalytic domain, and motif-I forms a short β-sheet with a positively charged surface near the active site. Circular dichroism spectra showed that residues 1–94 are in a random coil conformation. Deletion of the three additional fragments reduced the activity and thermal stability, compared to full-length mvUNG. The results suggested that the mvUNG N-domain and motif-I are required for its structural and functional integrity.

## Introduction

Although uracil is a base component of RNA, it is occasionally introduced into DNA by hydrolytic deamination of cytosine [1]. Uracil generated by cytosine deamination causes a ‘G to A mutation’ in the complementary strand during DNA replication, which must be corrected to maintain genomic integrity [1]. Uracil in DNA can be repaired by the base excision repair (BER) pathway [2, 3]. In the BER pathway, uracil-DNA glycosylase (UDG) recognizes uracil which exists in DNA and creates an abasic site by cleaving the N-glycosidic bond between uracil and deoxyribose. Apurinic/apyrimidinic (AP) endonuclease then removes the sugar-phosphate backbone of the abasic site. Finally, DNA polymerase adds a correct nucleotide to the site [2, 3].

UDG genes are found ubiquitously in cellular organisms from prokaryotes to humans, and have also been identified in some large DNA viruses, such as poxviruses and herpesviruses [2, 4, 5]. UDG proteins are classified into six families, based on characteristic motifs related to their substrate specificity and catalytic activity [6]. The family-I UDGs, called UNGs, are characterized by five conserved motifs that are arranged around the active site [7]. The motifs include the catalytic water-activating loop, the Pro-rich loop, the uracil-binding motif, the Gly-Ser loop, and the minor groove intercalation loop, corresponding to 143-GQDPYH-148, 165-PPPPS-169, 201-LLLN-204, 246-GS-247, and 268-HPSPLS-273 in human UNG1 (hUNG1), respectively [7–9].

Although UNG removes uracil included in single-stranded DNA (ssDNA) or double-stranded DNA (dsDNA), it shows a preference for ssDNA than dsDNA as a substrate [10, 11]. The catalytic mechanism of UNG has been studied extensively through structural and biochemical analyses [12–16]. It is described as “pinch, push, plug, and pull”. In human UNG, the Pro-rich, Gly-Ser, and minor groove intercalation loops lead to DNA bending by compressing the DNA backbone (pinch). Next, a Leu residue in the minor groove intercalation loop penetrates into the DNA double helix (push), resulting in the base flipping of uracil, followed by uracil insertion into the binding pocket of the active site (plug). After removing uracil from DNA, the Leu in the minor groove intercalation loop is retracted (pull).

The UNG gene in *Acanthamoeba polyphaga* mimivirus (APMV) was identified recently by its genome sequencing [17]. APMV, which belongs to the *Mimiviridae* family of nucleocytoplasmic large DNA viruses (NCLDV), is one of the largest known viruses, comparable to bacteria in size [18]. It has a large-sized virion (~750 nm) and harbors a complex genome (~1.2 Mb) that encodes approximately 1,000 putative genes. Some of these genes have been identified as core genes conserved among NCLDVs, whereas some are unique to cellular organisms and have never been identified in other viruses, blurring the boundary between cellular organisms and viruses [17, 19, 20]. Interestingly, APMV UNG (mvUNG) is longer than other known UNGs, suggesting that it contains domains or motifs in addition to the canonical catalytic domain. As a first step towards understanding of mvUNG, we determined its crystal structure and analyzed its enzymatic activity and thermal stability. The results suggested that the additional segments are required for mvUNG’s structural and functional integrity.

## Materials and methods

### Plasmid preparation, protein expression, and purification

The gene encoding mvUNG (residues 1–370; GenBank accession number AAV50521.1) was synthesized artificially after codon optimization (Cosmo Genetech, Seoul, South Korea) and inserted into pET-22b (Merck Millipore, Billerica, MA, USA) with DNA encoding His_6x_-thioredoxin. The plasmid was then transformed into *Escherichia coli* strain BL21-star(DE3) (Thermo Fisher Scientific, Waltham, MA, USA), and the cells were grown in Luria-Bertani medium. Protein expression was induced using 0.4 mM isopropyl-β-D-thiogalactoside at 15°C. After overnight induction, cells were harvested by centrifugation, and clarified cell lysates were prepared in buffer A (20 mM hydroxyethyl piperazineethanesulfonic acid (HEPES) pH 7.5, 0.5 M NaCl, 5% glycerol, and 0.2 mM tris(2-carboxyethyl)phosphine). The mvUNG protein was purified using immobilized metal affinity chromatography (IMAC) and size exclusion chromatography (SEC). Proteins purified by IMAC were dialyzed twice in buffer A and then treated with thrombin to cleave the His_x6_-thioredoxin tag from mvUNG. After complete cleavage of the tag, the protein solution was passed through IMAC resin to remove His_6x_-thioredoxin. mvUNG was further purified using a Superdex-75 size exclusion column equilibrated with buffer A, followed by a Superdex-200 column equilibrated with buffer C (20 mM HEPES pH 7.5, 0.2 M NaCl, 5% glycerol, and 0.2 mM tris(2-carboxyethyl)phosphine). Purified mvUNG was concentrated to 10 mg/mL and estimated to be >95% pure by SDS-PAGE. Mutant proteins (mvUNG_95-370_, mvUNG_122-370_, and mvUNG_Δ327–343_) were expressed and purified by the same procedure as that used for native mvUNG.

### Crystallization, data collection, and structure determination

Crystallization of mvUNG was performed using the micro-batch method at 20°C. The crystallization drop was prepared by mixing 1 μL of protein solution (10 mg/mL) and 1 μL of crystallization solution (0.1 M HEPES pH 7.5, 25% (v/v) Polyethylene glycol 3350, 4% (v/v) Isopropanol, and 0.1 M CaCl_2_) under a layer of Al’s oil (Hampton Research, Aliso Viejo, CA, USA). Needle-shaped crystals of mvUNG were fully grown in 2 months. Crystals were picked up using a cryo-loop (Hampton Research) and then flash-frozen in a cold nitrogen stream without the addition of a cryoprotectant. Diffraction data were collected at PLS-BL7A (Beam line 7A, Pohang Light Source, South Korea) and were indexed, integrated, and scaled using HKL2000 software [21].

The crystal structure of mvUNG was determined by the molecular replacement (MR) method using PHASER [22]. The structure of *Leishmania naiffi* UDG (PDB ID: 3CXM) was used as a template for MR. Two mvUNG proteins were found in an asymmetric unit. Cycles of refinement and model rebuilding were performed at 2.3 Å resolution using phenix.refine [23] and COOT [24]. Residues 95–370 in mvUNG were traced in the electron density. Final refinement with solvents resulted in R/R_free_ values of 17.3 / 21.6% without residues in the disallowed region of the Ramachandran plot. Data collection and refinement statistics are summarized in Table 1. The figures were drawn using PyMOL [25, 26] and ALSCRIPT [27]. Structural alignment was analyzed using the DALI server [28].

**Table 1:** Data collection and refinement statistics of mvUNG.

**Data collection**		
Space group		C222_1_
Unit cell		
	a, b, c (Å)	96.27, 95.62, 132.38
	α, β, γ (°)	90.00, 90.00, 90.00
Resolution (Å)		50.0–2.30 (2.34–2.30)
Wavelength (Å)		0.97934
Unique reflections		27418 (1370)
Redundancy		7.2 (7.3)
Completeness (%)		99.9 (100.0)
I/σ		26.4 (5.3)
Rmerge (%)		11.9 (57.3)
**Refinement**		
Resolution		30.0–2.30
No. reflections, working/free		26005/1376
R_work_/R_free_ (%)		17.3/21.6
No. atoms		
	Protein	4494
	Water	320
B factors		
	Protein	28.9
	Water	32.2
RMSD		
	Bond length (Å)	0.008
	Bond angle (°)	0.987
Ramachandran plot (%)		
	Favor	97.6
	Allowed	2.4
	Disallowed	0.0

### Activity assay of mvUDG

The activity of mvUNG was measured using ssDNA and dsDNA containing a single uracil (5’-GTA AAA CGA CGG CCA GTG UAT TCG AGC TCG GTA CCC GGG G-3’) as a substrate (Cosmo Genetech) [29]. The reaction mixture, containing 10 μM DNA substrate, 20 mM Tris-HCl pH 8.0, 1 mM dithiothreitol, and 1 mM ethylenediaminetetraacetic acid, was incubated with mvUNG at 37°C. After incubation for 2 h, mvUNG was inactivated at 95°C for 5 min. Abasic sites generated by mvUNG were incised by treatment with 0.1 mM NaOH at 95°C for 5 min. The reaction products were analyzed by electrophoresis on 16.7% (w/v) polyacrylamide gels containing 7–8 M urea and 1× Tris-borate-ethylenediaminetetraacetic acid (Biosesang, Seongnam, South Korea) and the substrates were visualized by in-gel silver staining (Thermo Fisher Scientific) [30]. The quantity of the uncleaved substrate was calculated using GelAnalyzer software, and data were analyzed using Minitab 17 (Minitab Inc.). The assay for the activity comparison of mvUNG mutants was repeated seven times and the activity values of activity were averaged.

### SEC with multi-angle laser light scattering (SEC-MALLS)

To measure its molecular size, purified mvUNG was injected into a Superdex-200 analytical column connected to Purifier FPLC (GE Healthcare, Chicago, IL, USA) and the elution products were applied to inline miniDAWN TREOS MALLS and Optilab digital signal processing refractive index detectors (Wyatt Technology Corporation, Santa Barbara, CA, USA). Data were collected at 1.0 s intervals at a flow rate of 0.4 mL/min and were analyzed using the ASTRA V software package (Wyatt Technology Corporation).

### Circular dichroism (CD) measurement

CD spectra were obtained using the JASCO J-810 spectropolarimeter (Jasco, Tokyo, Japan). For far-UV CD spectra, 10 μM protein in a 1 mm cuvette was exposed to a wavelength range of 200–260 nm, and the data were recorded at a 1 nm intervals with a scanning speed of 5 nm/min. Three spectra from a protein sample were collected and averaged. The spectra were smoothed after subtraction of the buffer spectrum. For the thermal melting spectra, 10 μM protein in a 1 mm cuvette was exposed to a wavelength of 220 nm. The thermal melting curves were recorded at 0.5°C intervals in the temperature range of 20–90°C. Temperature was increased at a rate of 2°C/min.

### Accession number

The final coordinate and structure factor for mvUNG were deposited into the Protein Data Bank (PDB ID: **5X55**).

## Results

### Structure determination of catalytically active mvUNG

**Fig 1:**
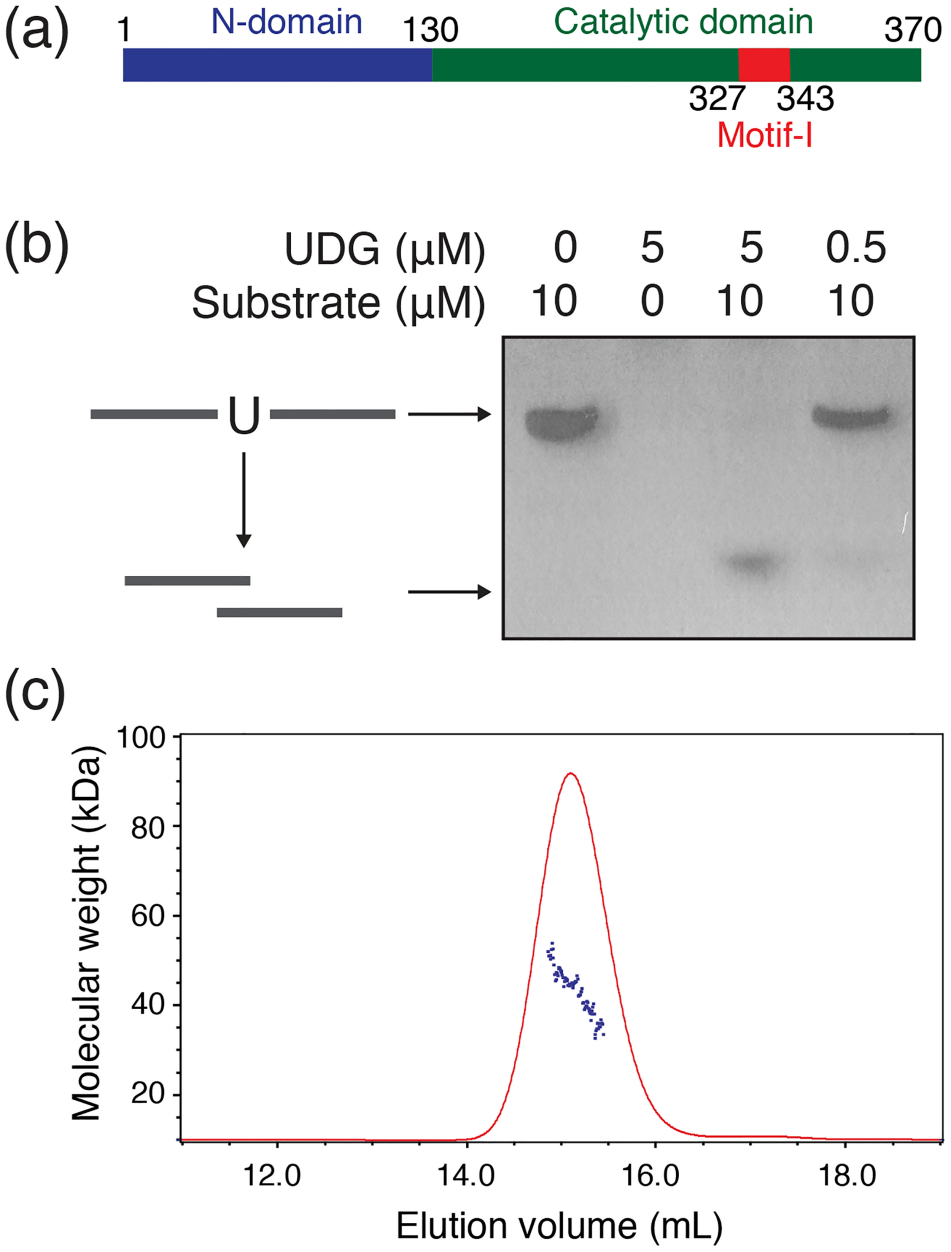
Characterization of mvUNG. (a) Overall domain organization of mvUNG. Green, blue, and red bars represent the relative lengths of the catalytic domain, N-terminal domain, and motif-I, respectively. The schematic shows that mvUNG contains a long N-terminal domain and motif-I, which is inserted in the catalytic domain. (b) Enzymatic activity of mvUNG. In the silver-stained gel, the top and bottom bands indicate uncleaved ssDNA containing a single uracil and cleaved fragments, respectively. This shows that mvUNG has the activity of uracil-DNA glycosylase activity. (c) Size measurement by SEC-MALLS. The molecular mass of purified mvUNG in solution was calculated as 43.4 kDa, which is close to that of the monomer (molecular weight of a monomer: 42.2 kDa).

mvUNG (residues 1–370) contains a catalytic domain conserved among UNG family proteins. Additionally, it has an N-terminal domain (N-domain; residues 1–130) and an insertional motif (motif-I; residues 327–343; Fig 1a). The N-domain in mvUNG is longer than those in other UNGs, and motif-I is not found in the catalytic domains of other known UNGs. For the structural and functional analysis of mvUNG, we prepared catalytically active mvUNG protein. The recombinant protein was expressed in *E*. *coli* and purified by Ni-affinity chromatography and SEC. The activity of the purified mvUNG was measured by incubating it with ssDNA or dsDNA containing a single uracil. In the assay, the incubated mixture was treated with NaOH to cleave the abasic site in DNA, and the cleavage was then visualized using a silver-stained gel. The gel showed that ssDNA substrate was cleaved in the presence of mvUNG, whereas dsDNA was not (Fig 1b and S1 Fig). This result indicates that the uracil in ssDNA is removed by mvUNG, while uracil in dsDNA does not seem to be a preferred substrate.

Needle-shaped crystals were grown from full-length mvUNG, and the diffraction data were collected up to 2.3 Å resolution. The phase was obtained by molecular replacement, using the structure of *Leishmania naiffi* UDG, which shows high sequence identity with the catalytic core domain of mvUNG. Residues 95–370 of two mvUNG proteins in the asymmetric unit were traced into the electron density (S2 Fig), and the final model was refined with R/R_free_ values of 17.3/21.6% (Table 1). Matthews’s coefficient (V_m_) and the solvent content under crystal packaging of full-length mvUNG were calculated as 1.8 Å^3^/Da and 31.8%, respectively, which are out of the range commonly observed for protein crystals, while those of mvUNG_95-370_ were 2.4 Å^3^/Da and 48.8%, which falls within the range. A solvent space that could accommodate residues 1–94 was insufficient in the crystal. Moreover, mvUNG_95-370_ facilitated the growth of the needle-shaped crystal under same crystallization conditions as full-length mvUNG. Thus, the N-terminal residues seem to be truncated before crystallization.

Two mvUNGs in the asymmetric unit were superimposed with a root-mean-square deviation (RMSD) of 0.4 Å for 276 Cα atoms, indicating that the two mvUNGs are in the same conformation. The oligomeric state in solution was determined by SEC. In SEC, mvUNG (molecular weight of a monomer: 42.2 kDa) was eluted as a monodisperse protein between ovalbumin (44 kDa) and conalbumin (75 kDa), which means that it was slightly larger size than a monomer of mvUNG (S3 Fig). To measure its oligomerization state precisely, mvUNG was applied to SEC-MALLS. The molecular mass of mvUNG calculated by SEC-MALLS was approximately 43.4 kDa (Fig 1c), indicating that the protein is a monomer in solution. Thus, the slightly faster elution in SEC seems to have resulted from a flexible region or an elongated shape of mvUNG.

### Overall structure of mvUNG

**Fig 2:**
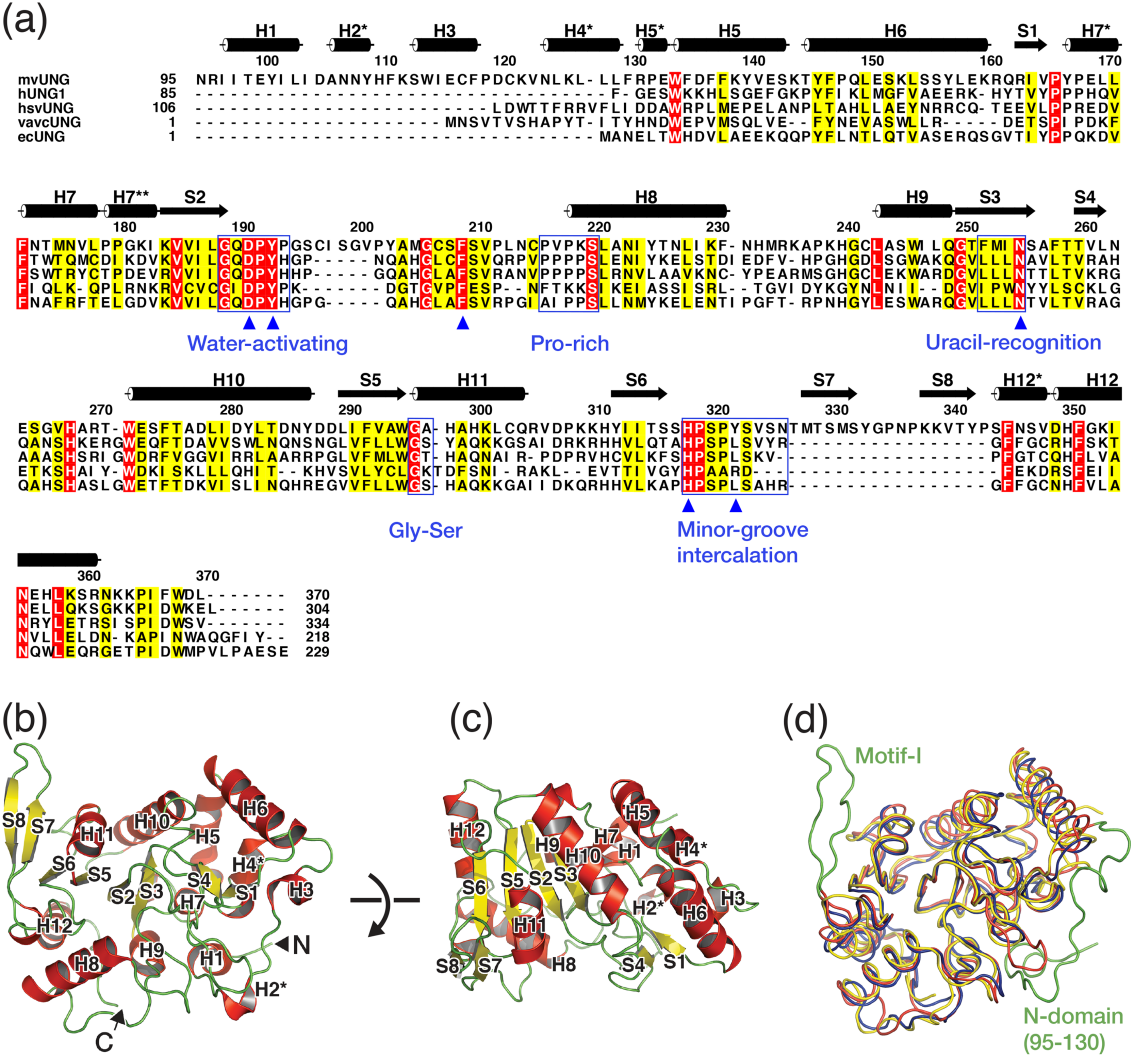
The structure of mvUNG. (a) Structure-based sequence alignment of UNGs. UNG sequences from *Acanthamoeba polyphaga* mimivirus (mvUNG), human (hUNG), herpes simplex virus (hsvUNG), vaccinia virus (vacvUNG) and *Escherichia coli* (EcUNG) are aligned based on a structural comparison. Identical and homologous residues are boxed in red and yellow, respectively. Residues of the active site are marked with blue triangles. The five conserved motifs in UNGs are boxed and labeled in blue. The secondary structure of mvUNG is displayed using a cylinders for helices and arrows for β-strands. Helices and β-strands are labeled with H and S, respectively. Helix labels with and without an asterisk (*) indicate an α-helix and 3_10_ helix, respectively. (b, c) Ribbon models of mvUNG shown at two different orientations. α-helix, β-strand, and loops are colored differently, and the secondary structures are labeled using the same scheme as in (a). (d) Structural comparison of UNGs. Structures of mvUNG, hUNG (PDB ID: 1AKZ), and hsvUNG (PDB ID: 1UDG) were superimposed, and Cα trace models were drawn in the same orientation as (b). Red, yellow, and blue represent mvUNG, hUNG, and hsvUNG, respectively. Additional segments in mvUNG (motif-I and residues 95–130 of the N-domain) are colored green and labeled.

Crystal structures of UNG proteins from diverse organisms and large viruses including human [8], *E*. *coli* [31], herpesviruses [32], and poxviruses [33] have been reported. Their catalytic domains share a highly conserved α/β fold containing a four-stranded parallel β-sheet surrounded by eight α-helices. The catalytic domain of mvUNG also forms a similar fold, comprising a central β-sheet (S2, S3, S5, and S6 Figs) sandwiched by α-helices (H5–H12). The N-termini of H5, H7, and H12 and the C-terminus of H7 extend to the 3_10_-helix. Additionally, two short β-strands (S1 and S4 Figs) form an antiparallel β-sheet, separately from the central β-sheet (Fig 2). Searching for structural similarity using the DALI program, showed that mvUNG has high similarity to UNG proteins, such as human UNG (hUNG) [14] and herpesvirus UNG (hsvUNG) [32]. hUNG and hsvUNG were superimposed onto mvUNG with RMSD values of 1.2 Å for 220 Cα and 1.6 Å for 221 Cα positions, respectively, which indicated that the catalytic domain of mvUNG shares a conserved fold with other UNG proteins (Fig 2d).

**Fig 3:**
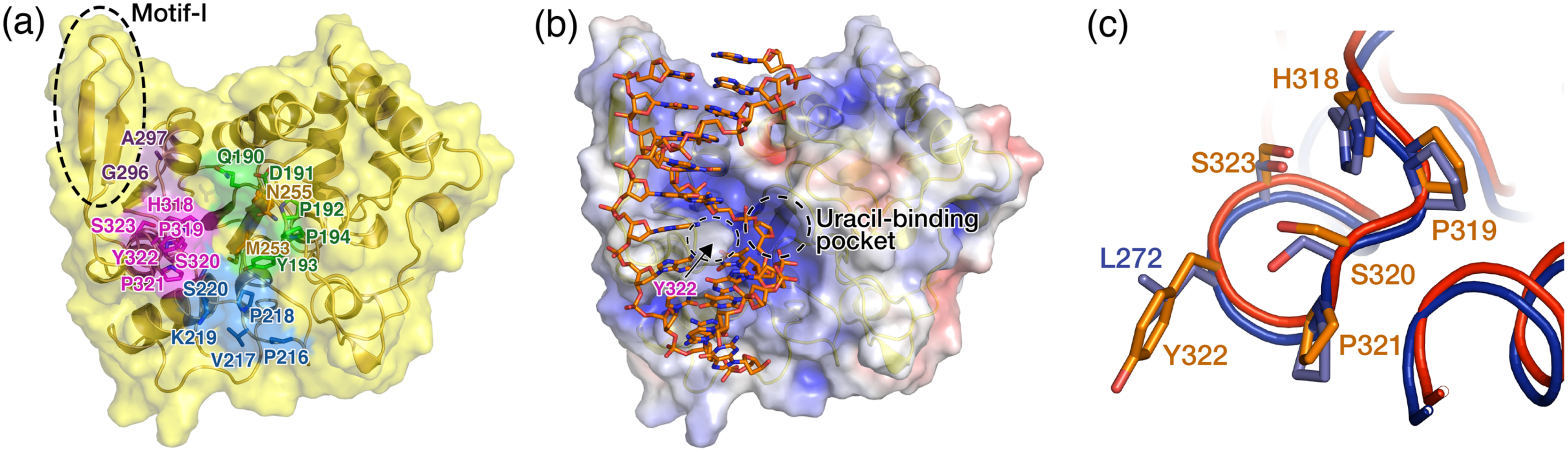
The active site of mvUNG. (a) Motifs of mvUNG conserved in UNG proteins. The ribbon and stick model covered by a transparent yellow surface is shown. Residues of the water-activating, Pro-rich, uracil-recognition, Gly-Ser, and minor-groove intercalation motifs are labeled and colored green, blue, orange, purple, and magenta, respectively. Motif-I (black dotted oval) is on the left side of the Gly-Ser loop (purple) and the minor-groove intercalation loop (magenta) in this figure. (b) The DNA-binding model of mvUNG. The model was generated by superimposing mvUNG on the structure of hUNG/DNA containing an abasic site (PDB ID: 2SSP). mvUNG was drawn as a surface model with charge distribution. DNA in the structure of hUNG/DNA complex is drawn as a stick model. The figure shows that DNA is intercalated by Y322 and binds to a positively charged groove formed by five motifs conserved in UNG family proteins. mvUNG structures in (a, b) are in the same orientation as in Fig 2b. (c) Comparison of active site residues. Structures of mvUNG and hUNG (PDB ID: 1AKZ) are superimposed. Red and blue Cα trace models represent mvUNG and hUNG, respectively. Residues of the active site are drawn as sticks and labeled. Leu residue in minor-groove intercalation loop of hUNG is replaced by Tyr (Y322) in mvUNG.

In addition to the canonical fold of UNGs, the structure of mvUNG contains a long N-terminal domain (N-domain; residues 1–130) and an insertional motif (motif-I; residues 327–343) located between S6 and H12 (Fig 2). The N-domains in UNG proteins are highly variable in length and do not share conserved motifs. In the structure of mvUNG, residues 95–130 comprise four short helices, two α-helices (H1 and H3), and two 3_10_-helices (H2 and H4). Residue R96 in the N-domain forms an ionic interaction with E169 in the catalytic domain, and the segment is fitted into a hydrophobic groove of the catalytic domain. Residues 95–130 of the N-domain seem to stabilize the catalytic core domain by binding to its hydrophobic surface (S4 Fig). Motif-I forms a two-stranded antiparallel β-sheet (S7 and S8) (Figs 2 and 3a) and provides a positively charged surface near the active site, which might improve the interaction with the DNA backbone (S5 Fig). Taken together, the mvUNG structure suggests that the visible N-domain (residues 95–130) and motif-I (residues 327–343) might affect the structural and functional integrity of mvUNG.

### The active site of mvUNG

UNG proteins from human, *E*. *coli*, and herpesviruses contain six conserved active site residues that are involved in uracil-binding specificity and catalysis (Fig 2a; D145, Y147, F158, N204, H268, and L272 in human UNG1) [8, 31, 32]. However, some UNG proteins do not contain a Leu residue in the minor groove intercalation loop [33, 34]. For example, the Leu residue is replaced by hydrophobic Phe in *Bacillus subtilis* UNG and by positively charged Arg in vaccinia virus UNG (vacvUNG). mvUNG also does not contain the Leu residue (Fig 2a). The catalytic residue (D191) and the other four residues for uracil-binding specificity in the binding pocket (Y193, F209, N255, and H318) are conserved as the canonical active site residues, whereas Leu in the minor groove intercalation loop is replaced by Y322 in mvUNG (Figs 2a and 3c). The bulky polar residue, Y322, does not induce a conformational change of the minor groove intercalation loop. The loop conformation, including amino acid side chains, is superposed well with that of human UNG (Fig 3c). The Leu residue (Phe in *B*. *subtilis* UNG) in the minor groove intercalation loop is a critical residue for the inhibition of UNGs by Ugi and p56, as well as for catalytic activity. The Leu or Phe residue is recognized by the hydrophobic pockets of Ugi [12, 35–37] and p56 [34]. The vacvUNG, which contains Arg instead of Leu in the minor groove intercalation loop, is not inhibited by Ugi [38]. This implies that the interaction between mvUNG and inhibitor proteins (Ugi and p56) might be prevented or weakened by the hydroxyl group of Y322 in mvUNG.

In addition to active site residues, UNGs share five conserved motifs required for the formation of a uracil-binding pocket and DNA-binding groove (Figs 2a and 3). Conserved motifs include the Pro-rich and Gly-Ser loops for the compression of DNA substrate, the minor groove intercalation loop (or Leu-intercalation loop) for the separation of ds DNA, and the uracil-recognition and water-activating loops for enzymatic catalysis. In mvUNG, 189-GQDPYP-194, 216-PVPKS-220, 252-FMIN-255, 296-GA-297, and 318-HPSPYSVSN-326 are structurally aligned as the water-activating, Pro-rich, uracil-recognition, Gly-Ser, and minor-groove intercalation loops, respectively (Figs 2a and 3). In the comparison of the conserved motifs, 16 and 11 out of 26 residues in the motifs of mvUNG are identical to those of hUNG and vacvUNG, respectively (Fig 2a).

### Deletions of N-domain and motif-I reduce enzymatic activity

**Fig 4:**
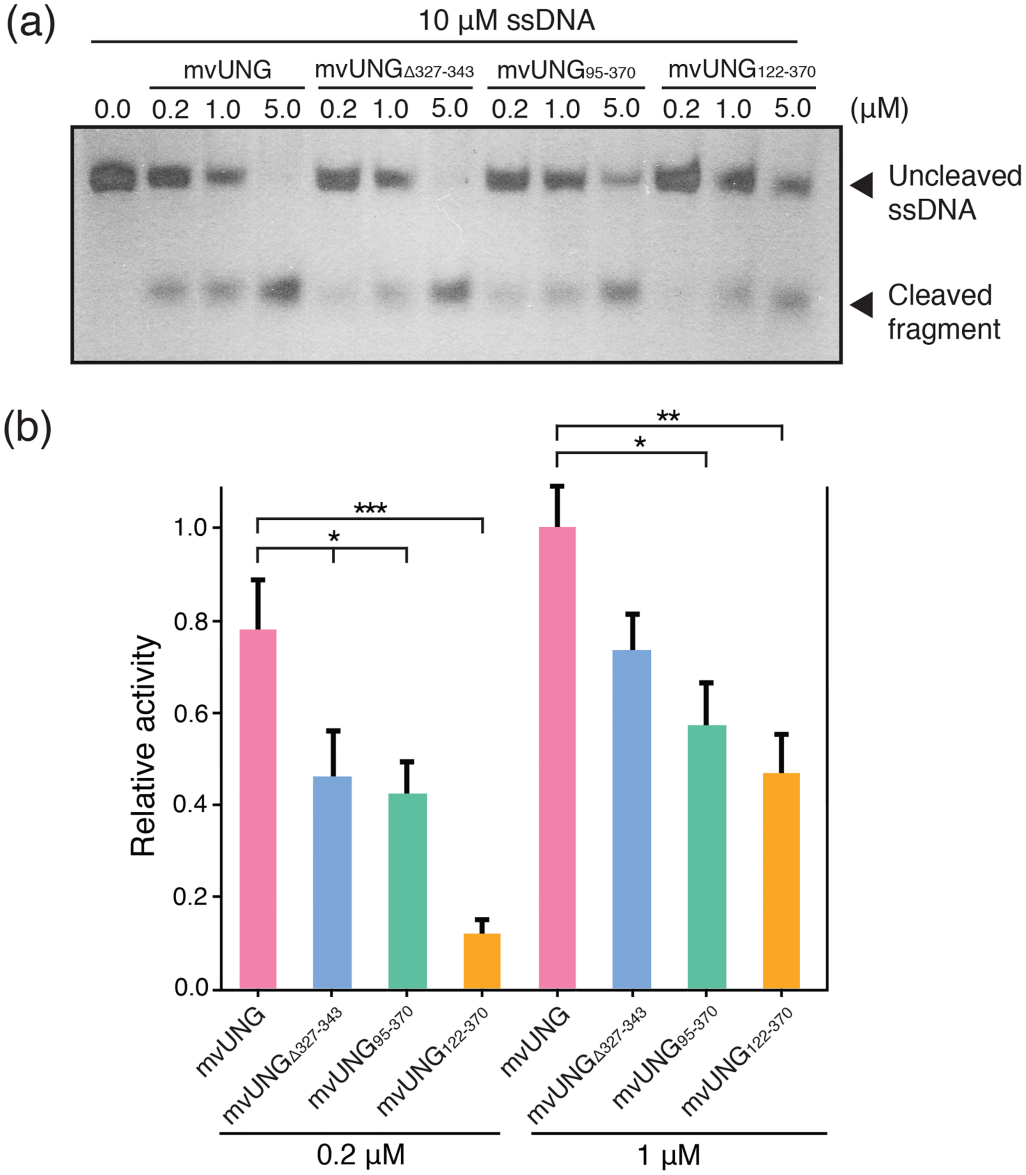
Activity comparison of mvUNG mutants. (a) Silver-stained gel showing the cleavage of ssDNA by mvUNG mutants. Full-length mvUNG, mvUNG_Δ327–343_, mvUNG_95-370_, and mvUNG_122-370_ were incubated with ssDNA to compare their UNG activities. (b) Relative activities of mvUNG mutants drawn as a histogram. The activity was calculated based on the amount of remaining uncleaved substrate. Seven independent measurements were averaged (S6 Fig). *P* values were calculated using a two-sample *t*-test. Error bars indicate mean ± standard error (*P< 0.05, **P < 0.01, ***P < 0.001, n = 7). In the reaction of 0.2 μM mvUNG and 10 μM ssDNA, the activities of mvUNG_Δ327–343_, mvUNG_95-370_, and mvUNG_122-370_ were reduced to 59, 55, and 15% of the activity of full-length mvUNG, respectively. In the reaction of 1 μM mvUNG and 10 μM ssDNA, the activities of mvUNG_Δ327–343_, mvUNG_95-370_, and mvUNG_122-370_ were reduced to 73, 58, and 47% of the activity of the full-length mvUNG, respectively.

To evaluate the effects of the additional segments (residues 1–94, 95–130 and 327–343) on UNG activity, we performed activity assays with the mutant mvUNG proteins. A motif-I deletion mutant (mvUNG_Δ327–343_) and two N-terminal deletion mutants (mvUNG_95-370_ and mvUNG_122-370_) were prepared using the same procedure as the full-length mvUNG. The activity assays showed that UNG activity of the three deletion mutants was significantly lower than that of the full-length mvUNG (Fig 4).

Motif-I (residues 327–343) of mvUNG is located around the Gly-Ser and minor-groove intercalation loops, without disrupting the active site. Its surface distributes a weakly positive charge (Fig 3 and S5 Fig). In the activity assay, the deletion of motif-I reduced the activity of mvUNG to 59% in the reaction of 0.2 μM mvUNG_Δ327–343_ and 10 μM ss-DNA substrate, compared with the activity of full-length mvUNG (first and second bars in Fig 4b). This implied that motif-I might affect the efficiency of the active site by improving the interaction between the enzyme and DNA. The N-domain in eukaryotic and viral UNGs varies in length and its sequences are not conserved. It seems to contribute to diverse cellular functions of UNGs. For example, the N-domain of hUNG is involved in the subcellular localization of isoforms [39] and the interaction with the DNA repair factor RPA [40, 41]. In the activity assay of the N-domain deletion mutants, mvUNG_95-370_ which residues 1–94 is deleted showed 55% activity, compared with full-length mvUNG (first and third bars in Fig 4b). Additional deletion of residues 95–121 reduced the activity to 15%, even though the deletion mutant contains the entire catalytic domain (first and fourth bars in Fig 4b). This result indicated that both invisible (residues 1–94) and visible (residues 95–130) segments of the N-domain are required for the intrinsic activity of mvUNG.

### The flexible region of the N-domain (residues 1–94) affects the thermal stability of mvUNG

**Fig 5:**
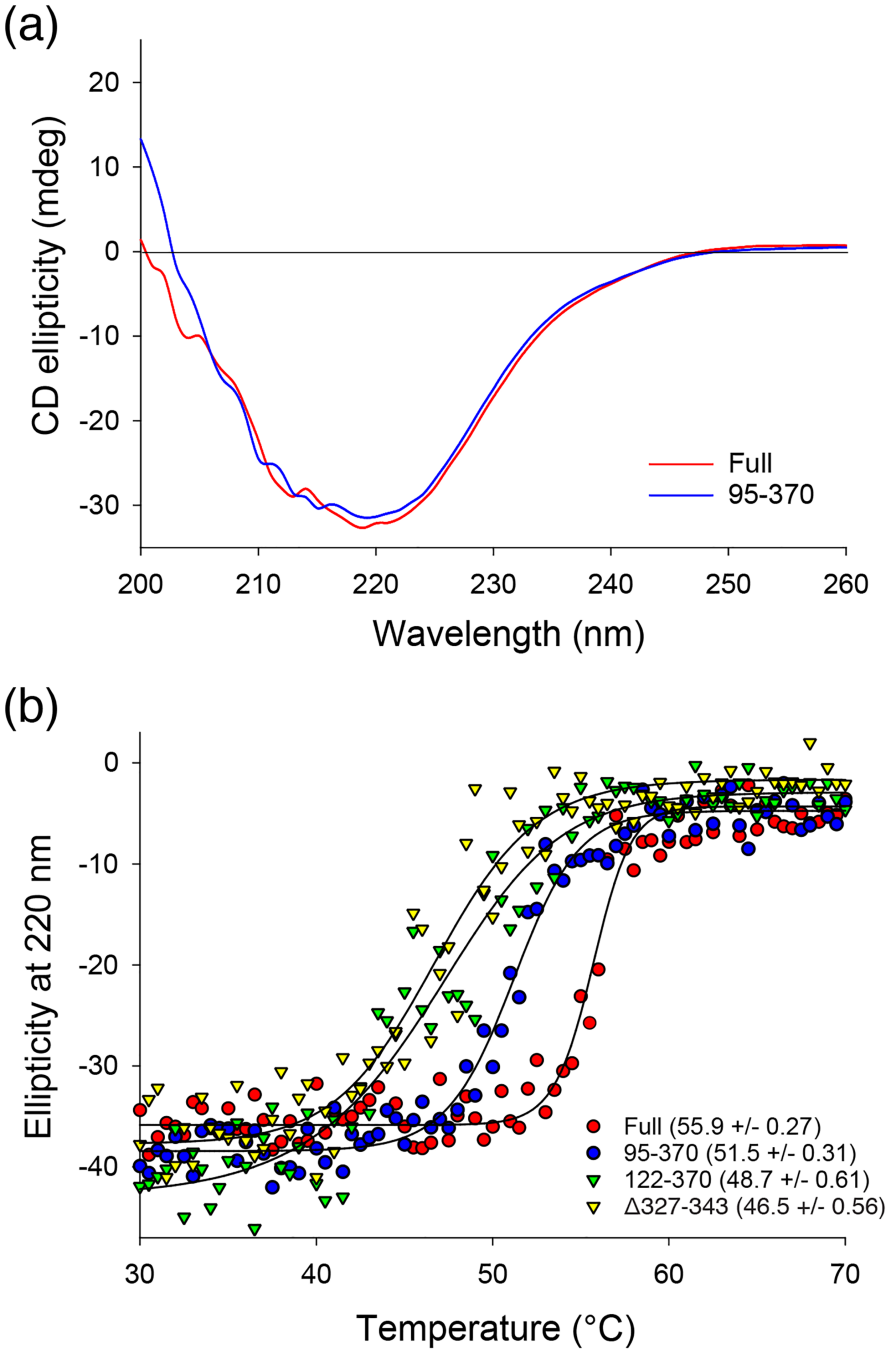
Circular dichroism (CD) spectra of mvUNGs. (a) Far-UV CD spectra of full-length mvUNG (Full) and mvUNG_95-370_ (95–370). The two CD spectra show similar patterns and ellipticity scales, indicating that residues 1–94 of mvUNG are close to a random coil conformation. (b) Thermal melting curves. Melting curves were collected at a wavelength of 220 nm by increasing temperature to 90°C. Calculated T_m_ values of full-length mvUNG, mvUNG_95-370_, mvUNG_122-370_, and mvUNG_Δ327–343_ were 55.9, 51.5, 48.7, and 46.5°C, respectively, indicating that deletion of the additional segments decreased the thermal stability of mvUNG.

The N-domain is required for the intrinsic activity of mvUNG, as shown above (Fig 4). Residues 95–130 appear to affect the catalytic domain by binding to its hydrophobic surface, while residues 1–94 are not visible in the crystal structure of mvUNG, probably due to its truncation before crystallization. To evaluate the secondary structure of the invisible segment in the N-domain, we compared the CD spectra of mvUNG and mvUNG_95-370_. In the far-UV CD spectra, the deletion of residues 1–94 did not change the pattern and scale of the CD spectrum in the range between 210–250 nm, indicating that residues 1–94 have a random coil conformation (Fig 5a). Consistent with the CD data, residues 1–40 and 50–90 of mvUNG were predicted to be disordered regions using a computational approach. Next, CD ellipticity at 220 nm was measured by increasing the temperature gradually from 20 to 90°C to calculate melting temperature (T_m_). The T_m_ value of full-length mvUNG (55.9°C) was 4.4°C higher than that of mvUNG_95-370_ (51.5°C) (Fig 5b), indicating that the deletion of residues 1–94 decreases the thermal stability of mvUNG. Activity assays and CD spectra implied that the unstructured segment (residues 1–94) in the N-domain also contributes to thermal stability and catalytic activity of mvUNG.

## Discussion

UNG is a ubiquitous enzyme for base excision repair. All UNGs, from human to large viruses, contain a conserved catalytic domain that removes uracil from DNA. Recently, a gene was identified from a mimivirus that encodes an UNG containing a long N-domain and motif-I in addition to the catalytic core domain [17]. To understand the structural features of the additional segments, we determined the crystal structure of mvUNG. In the crystal structure of mvUNG, residues 95–130 in the N-domain bind to the hydrophobic groove of the catalytic domain (S4 Fig). The deletion of residues 95–121 decreased the activity (Fig 4b) and thermal stability (Fig 5b) of the crystallized protein (mvUNG_95-370_). Thus, the structured part of the N-domain seems to stabilize the catalytic domain by binding directly to its hydrophobic surface. Motif-I, inserted into the catalytic domain, forms a short β-sheet near the active site (S5 Fig). The deletion of motif-I also decreased the activity (Fig 4b) and thermal stability of mvUNG (Fig 5b). The UNG activity of mvUNG_Δ327–343_ was similar to that of mvUNG_95-370_, while its thermal stability was lower than the N-domain deletion mutants. Although the structural features and activity assays indicated that motif-I might contribute to DNA binding, the possibility of reduced activity by decreased thermal stability cannot be excluded. Thus, the activity assay and thermal melting curves suggested that both the structured part of the N-domain (residues 95–130) and motif-I (residues 327–343) are required for the structural and functional integrity of mvUNG.

In contrast to the structured part of the N-domain and the motif-I, residues 1–94 in N-domain are not included in the crystal structure of mvUNG. They seem to be truncated before crystallization. Indeed, all reported crystal structures of UNG proteins display only a catalytic domain. CD spectra showed that residues 1–94 in mvUNG are close to a random coil (Fig 5a). Although the flexible fragment seems to be unnecessary for UNG activity and thermal stability of the core domain, the deletion of residues 1–94 in mvUNG decreased the activity and thermal stability (Figs 4 and 5). It is unclear how the flexible fragment affects the function of mvUNG. One possibility is an intramolecular interaction between the catalytic domain and the flexible N-domain. The intramolecular interaction might be not tight enough or might not be a specifically ordered interaction. Recently, it was reported that the disordered C-domain of human DNA glycosylase NEIL1 (hNEIL1) stabilizes the catalytic domain via electrostatic interactions [42, 43]. In a binding assay of hNEIL1, performed by His-tag pull-down followed by immunoblotting, the cluster of basic residues (355-KKGRRK-360) in an intrinsically disordered C-terminal domain weakly bound to the acidic cluster in the core domain. Notably, the N-domain of mvUNG also contains a cluster of basic residues (69-KKSKKSKKSKKSKKS-83), suggesting that the cluster might be associated with the integrity of mvUNG. Although the disordered domains between mvUNG and hNEIL1 are located at the opposite ends of amino acid sequences; they may function similarly because the N- and C-termini are exposed in the same direction. In addition, the N-domain of mvUNG and the C-domain of hNEIL1 share 15% sequence identity over about 100 amino acids, which is higher than that between the N-domains of other UNGs.

## Supporting information

S1 Fig Comparison of ssDNA and dsDNA as substrates for mvUNG. The UDG activity of mvUNG was measured using ssDNA and dsDNA as a substrate. Uracil was removed from ssDNA but not dsDNA.
(TIF)

S2 Fig Two mvUNG proteins in an asymmetric unit (MolA and MolB). (a) Ribbon model of two monomers in an asymmetric unit. (b) Cα trace model of two monomers superimposed.
(TIF)

S3 Fig Size exclusion chromatography of mvUNG. mvUNG was eluted as a monodisperse protein between ovalbumin (44 kDa; red triangle) and conalbumin (75 kDa; blue triangle) in both the Superdex-200 (a) and Superdex-75 (b) analytical columns.
(TIF)

S4 Fig The structure of the N-domain in mvUNG. (a) The visible N-domain and catalytic domain in mvUNG are drawn as stick and surface models, respectively. (b) The mvUNG monomer (residues 95–370) was drawn as a surface model with the charge distribution. (a, b) The panels show that the ordered fragment (residues 95–130) in the N-domain binds to the hydrophobic surface of the catalytic domain.
(TIF)

S5 Fig DNA-binding models of mvUNG (a) and mvUNG_Δ327–343_ (b). The models were generated by superimposing mvUNG and mvUNG_Δ327–343_ on the structure of hUNG/DNA containing an abasic site (PDB ID: 2SSP). mvUNGs and DNA were drawn as surface and backbone models, respectively. Motif-I (residues 327–343) does not disturb DNA-binding and provides a positively charged surface near the active site.
(TIF)

S6 Fig Silver-stained polyacrylamide gels used to calculate mvUNG activity. (TIF)
