# Supplementary figures and images for: Crystal structure of mimivirus uracil-DNA glycosylase
Source: PLoS One. 2017 Aug 1;12(8):e0182382. doi: 10.1371/journal.pone.0182382 (PMC5538708; doi:10.1371/journal.pone.0182382)

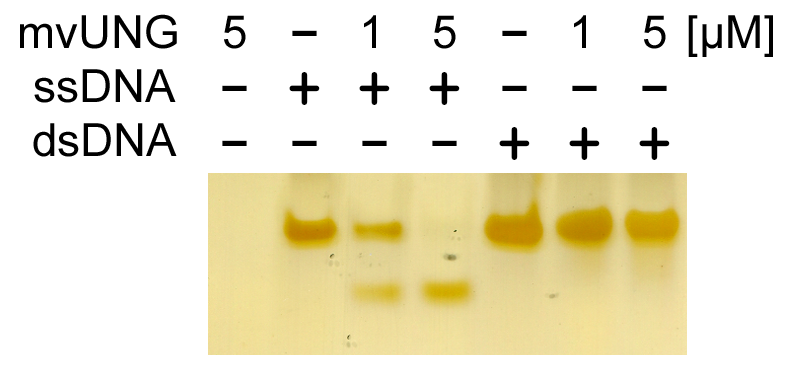

Supplement: S1 Fig — The UDG activity of mvUNG was measured using ssDNA and dsDNA as a substrate. Uracil was removed from ssDNA but not dsDNA. (TIF) [file pone.0182382.s001.tif]

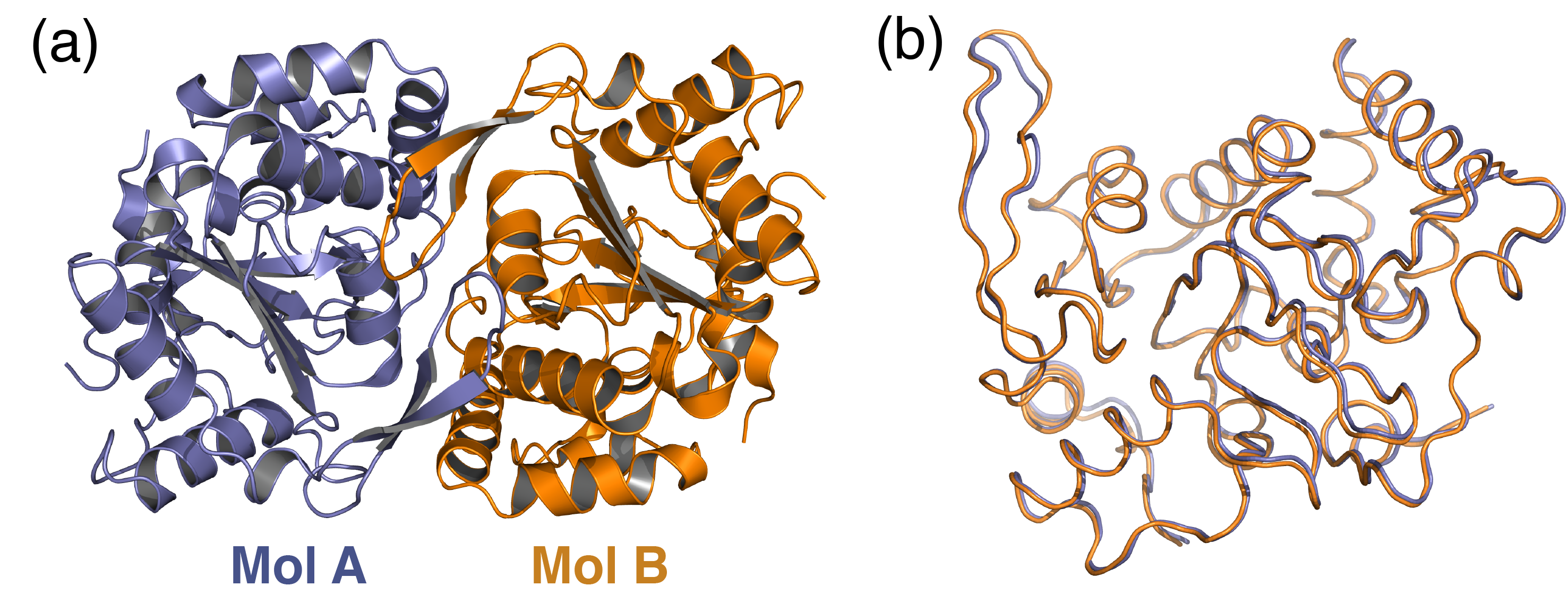

Supplement: S2 Fig — (a) Ribbon model of two monomers in an asymmetric unit. (b) Cα trace model of two monomers superimposed. (TIF) [file pone.0182382.s002.tif]

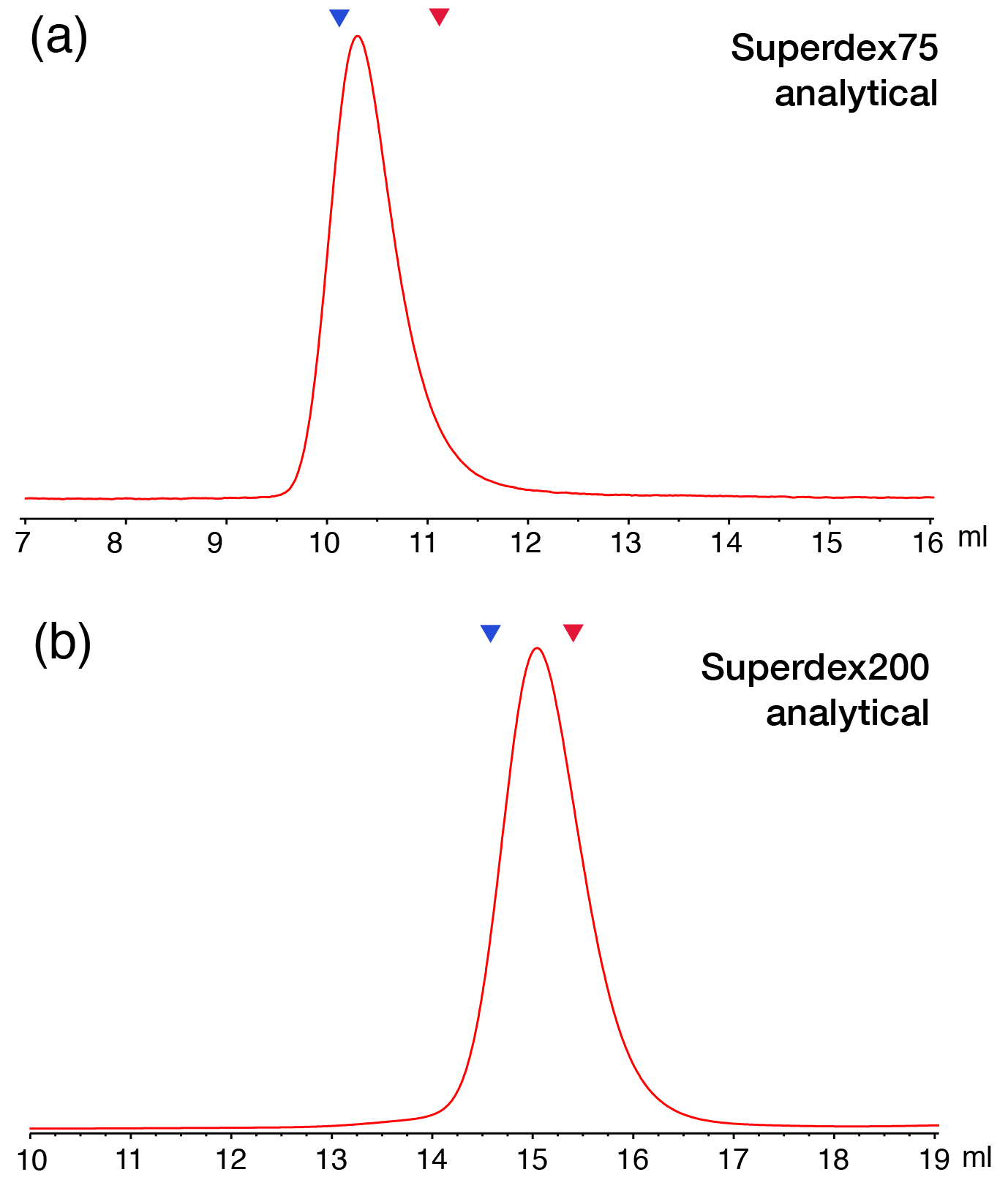

Supplement: S3 Fig — mvUNG was eluted as a monodisperse protein between ovalbumin (44 kDa; red triangle) and conalbumin (75 kDa; blue triangle) in both the Superdex-200 (a) and Superdex-75 (b) analytical columns. (TIF) [file pone.0182382.s003.tif]

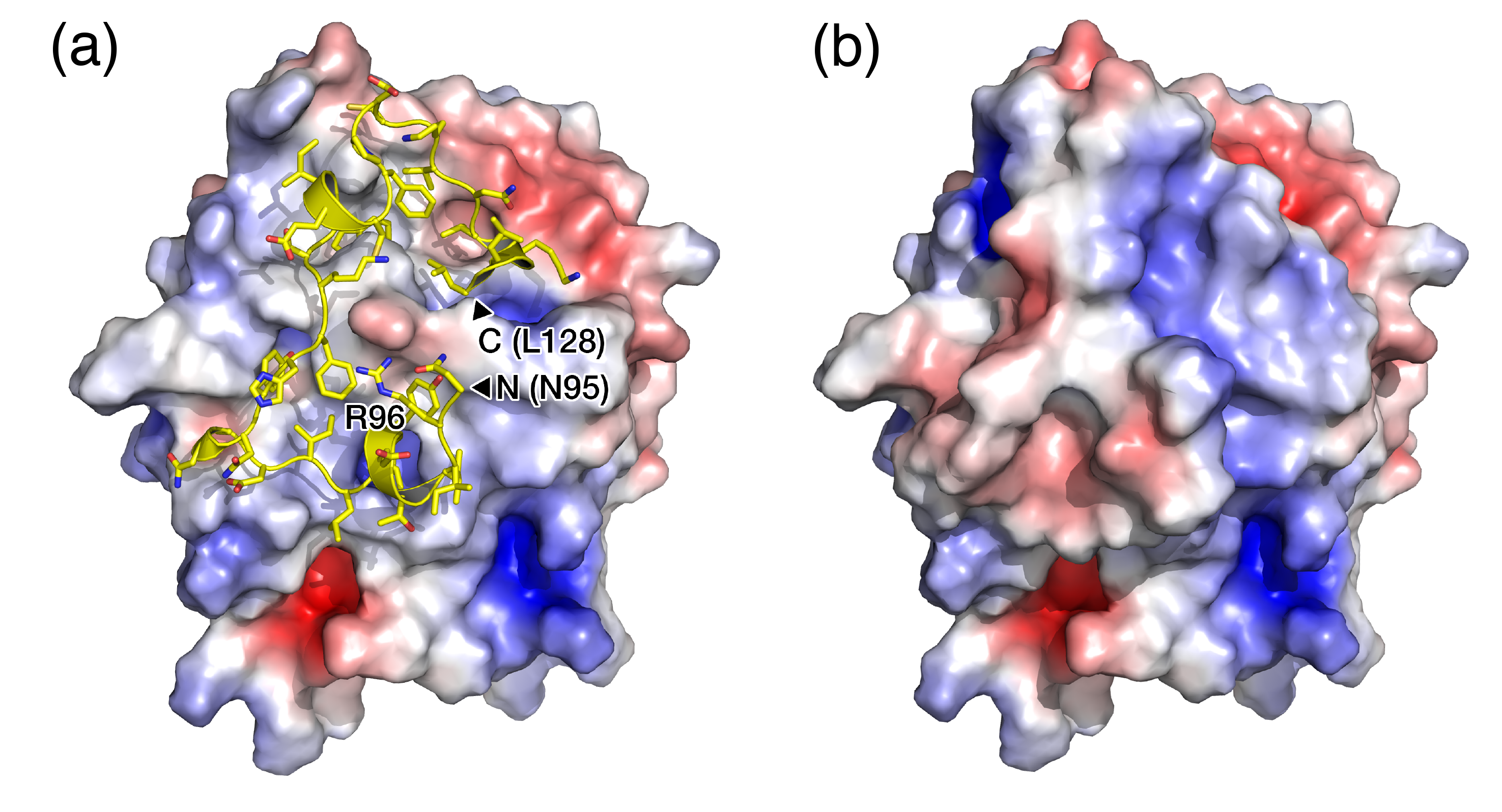

Supplement: S4 Fig — (a) The visible N-domain and catalytic domain in mvUNG are drawn as stick and surface models, respectively. (b) The mvUNG monomer (residues 95–370) was drawn as a surface model with the charge distribution. (a, b) The panels show that the ordered fragment (residues 95–130) in the N-domain binds to the hydrophobic surface of the catalytic domain. (TIF) [file pone.0182382.s004.tif]

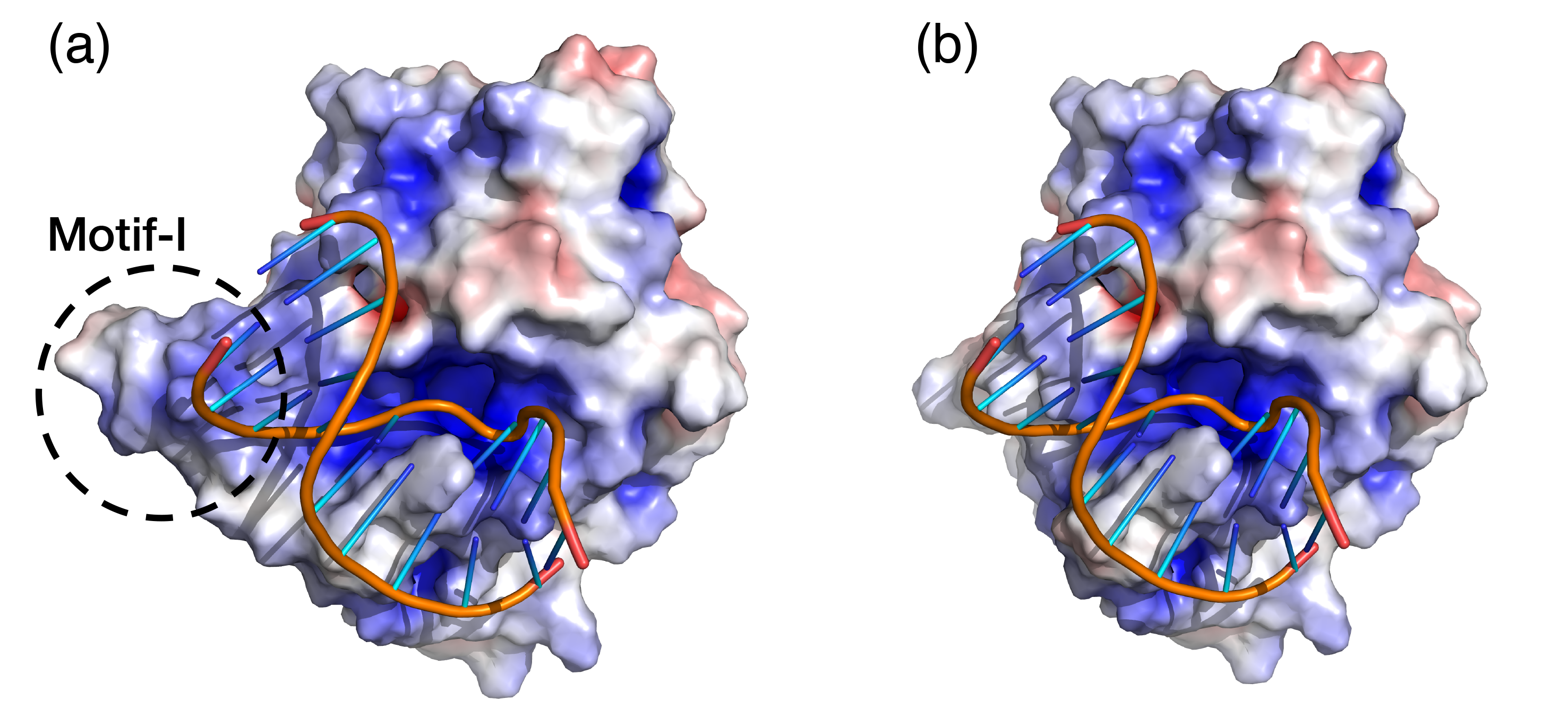

Supplement: S5 Fig — The models were generated by superimposing mvUNG and mvUNGΔ327–343 on the structure of hUNG/DNA containing an abasic site (PDB ID: 2SSP). mvUNGs and DNA were drawn as surface and backbone models, respectively. Motif-I (residues 327–343) does not disturb DNA-binding and provides a positively charged surface near the active site. (TIF) [file pone.0182382.s005.tif]

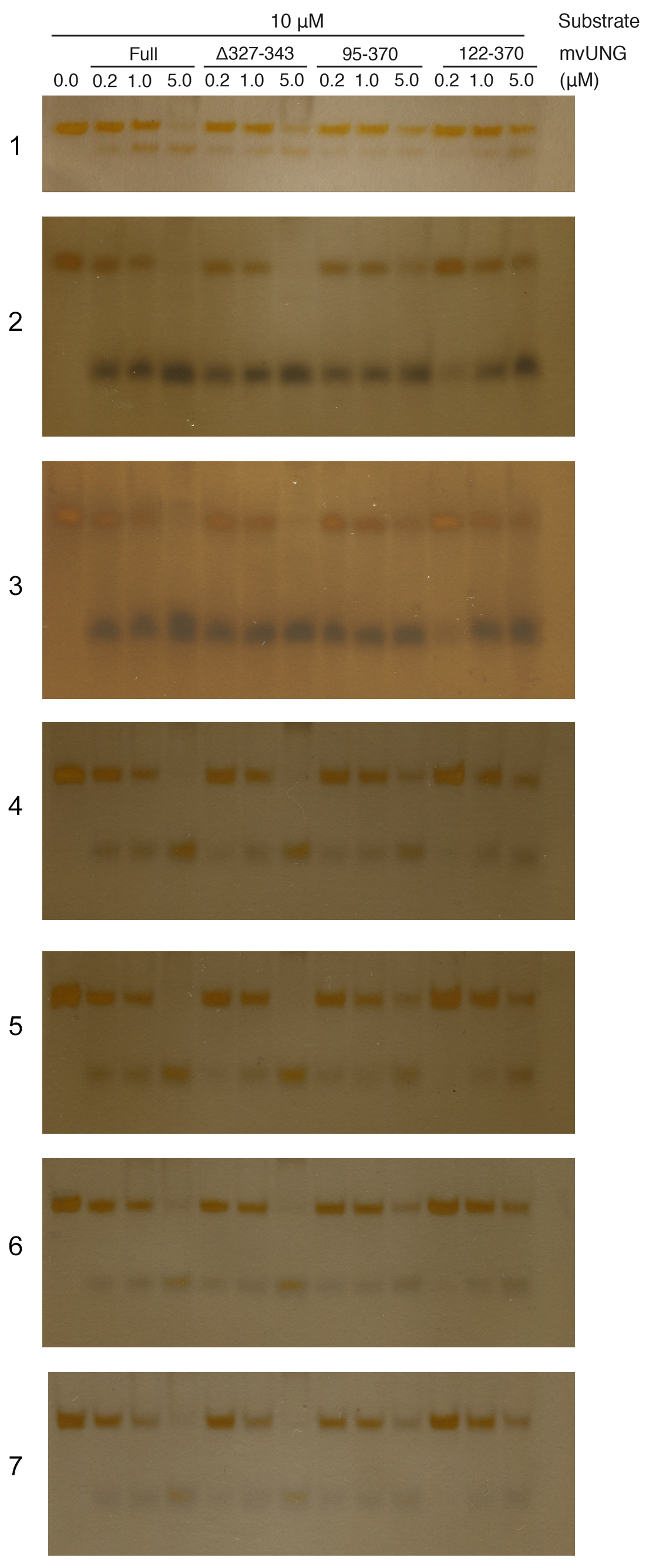

Supplement: S6 Fig — (TIF) [file pone.0182382.s006.tif]
